# Trends in genome-wide and region-specific genetic diversity in the Dutch-Flemish Holstein–Friesian breeding program from 1986 to 2015

**DOI:** 10.1186/s12711-018-0385-y

**Published:** 2018-04-11

**Authors:** Harmen P. Doekes, Roel F. Veerkamp, Piter Bijma, Sipke J. Hiemstra, Jack J. Windig

**Affiliations:** 1 Animal Breeding and Genomics, Wageningen University & Research, P.O. Box 338, 6700 AH Wageningen, The Netherlands; 2 Centre for Genetic Resources the Netherlands, Wageningen University & Research, P.O. Box 338, 6700 AH Wageningen, The Netherlands

## Abstract

**Background:**

In recent decades, Holstein–Friesian (HF) selection schemes have undergone profound changes, including the introduction of optimal contribution selection (OCS; around 2000), a major shift in breeding goal composition (around 2000) and the implementation of genomic selection (GS; around 2010). These changes are expected to have influenced genetic diversity trends. Our aim was to evaluate genome-wide and region-specific diversity in HF artificial insemination (AI) bulls in the Dutch-Flemish breeding program from 1986 to 2015.

**Methods:**

Pedigree and genotype data (~ 75.5 k) of 6280 AI-bulls were used to estimate rates of genome-wide inbreeding and kinship and corresponding effective population sizes. Region-specific inbreeding trends were evaluated using regions of homozygosity (ROH). Changes in observed allele frequencies were compared to those expected under pure drift to identify putative regions under selection. We also investigated the direction of changes in allele frequency over time.

**Results:**

Effective population size estimates for the 1986–2015 period ranged from 69 to 102. Two major breakpoints were observed in genome-wide inbreeding and kinship trends. Around 2000, inbreeding and kinship levels temporarily dropped. From 2010 onwards, they steeply increased, with pedigree-based, ROH-based and marker-based inbreeding rates as high as 1.8, 2.1 and 2.8% per generation, respectively. Accumulation of inbreeding varied substantially across the genome. A considerable fraction of markers showed changes in allele frequency that were greater than expected under pure drift. Putative selected regions harboured many quantitative trait loci (QTL) associated to a wide range of traits. In consecutive 5-year periods, allele frequencies changed more often in the same direction than in opposite directions, except when comparing the 1996–2000 and 2001–2005 periods.

**Conclusions:**

Genome-wide and region-specific diversity trends reflect major changes in the Dutch-Flemish HF breeding program. Introduction of OCS and the shift in breeding goal were followed by a drop in inbreeding and kinship and a shift in the direction of changes in allele frequency. After introduction of GS, rates of inbreeding and kinship increased substantially while allele frequencies continued to change in the same direction as before GS. These results provide insight in the effect of breeding practices on genomic diversity and emphasize the need for efficient management of genetic diversity in GS schemes.

**Electronic supplementary material:**

The online version of this article (10.1186/s12711-018-0385-y) contains supplementary material, which is available to authorized users.

## Background

Genetic variation in (closed) livestock populations is largely driven by the fundamental processes of selection and genetic drift. While selection acts directionally on alleles that have a selective (dis)advantage and on alleles that are ‘hitchhiking’ [1–3], genetic drift acts across the whole genome, causing random changes in allele frequency from generation to generation as a result of sampling gametes in a finite population [4].

In Holstein–Friesian dairy cattle (HF), intense artificial selection has been practised over many years. The use of a limited number of elite sires has reduced the effective population to a size ranging from 49 to 115 [5–7]. This implies that, in spite of its census size of millions of individuals, the breed is subjected to the same rate of genetic drift and accumulation of inbreeding as an idealized population of 49 to 115 individuals [4]. To ensure adaptive capacity and limit inbreeding depression in the long term, it is important to monitor and manage genetic diversity in the HF population [8, 9].

Traditionally, genetic diversity has been characterised and managed with pedigree-based coefficients of inbreeding and kinship, which refer to the proportion of the genome that is expected to be identical by descent (IBD) within and between individuals, respectively. However, this genealogical approach has several limitations: (1) it strongly depends on pedigree completeness and quality (e.g. [10]); (2) it does not account for Mendelian sampling variation (e.g. [11]); and (3) it only provides a genome-wide expectation for loci that are selection-free, i.e. loci that are in complete linkage equilibrium with all loci under selection (e.g. [12]).

With the wide availability of dense single nucleotide polymorphism (SNP) data, it has become possible to obtain more accurate estimates of genome-wide inbreeding and kinship and to evaluate diversity for specific regions of the genome [13–15]. Two approaches have been widely used to characterise and manage diversity from SNP data: the marker-by-marker approach [16] and the segment-based approach [17, 18]. The former approach involves the calculation of the observed and expected fraction of SNPs for which alleles are identical by state (IBS). Thus, it captures relationships that are caused by common ancestors going back to a very distant theoretical base population in which all alleles were unique. The second approach considers IBS segments, rather than individual SNPs. Since the length of these segments follows an inverse exponential distribution with expectation 1/2*G* Morgan [19], where *G* is the number of ancestral generations to the common ancestor from which the segment was derived, this approach may be used to distinguish recent from distant relatedness and move from IBS to ‘realised IBD’ [17]. Both IBS and IBD are relevant for management purposes. While IBS is the most direct diversity measure, (realised) IBD is more closely associated to inbreeding depression [18, 20, 21].

In recent decades, HF selection schemes have undergone profound changes with respect to inbreeding management, breeding goal composition and breeding value estimation. Around the year 2000, optimal contribution selection (OCS) was introduced to maximise genetic gain at a restricted rate of inbreeding [22]. Around the same time, national selection indices moved from production- and conformation-based only to more comprehensive indices that included traits related to production, conformation, longevity, health and reproduction [23]. More recently, genomic selection (GS) was introduced, which enabled the prediction of high-accuracy breeding values at a young age [24]. Since all these changes cause rearrangements in the ranking of artificial insemination (AI) bulls, they are expected to have influenced trends in genome-wide and region-specific genetic diversity. With the current availability of SNP-data, it is now possible to investigate this influence.

The aim of this study was to evaluate genome-wide and region-specific genetic diversity in HF AI bulls from 1986 to 2015, using genealogical, marker-by-marker and segment-based approaches. An important objective was to evaluate whether major changes in the Dutch-Flemish HF breeding program were accompanied by changes in inbreeding and kinship trends. A second objective was to investigate whether observed changes in allele frequency could be attributed to selection, and whether regions under selection could be linked to known quantitative trait loci (QTL). A last objective was to investigate how the direction of changes in allele frequency has evolved over time.

## Methods

### Animals and data

**Fig. 1:**
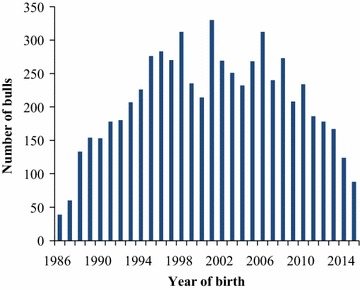
Number of genotyped bulls by year of birth

A total of 6280 AI bulls with breed fraction  higher than 87.5% HF, born between 1986 and 2015 and genotyped by the Dutch-Flemish cattle improvement co-operative (CRV), were included in this study. Thus, the vast majority of AI bulls in the Dutch-Flemish breeding program were included. Figure 1 shows the number of bulls by year of birth.

**Fig. 2:**
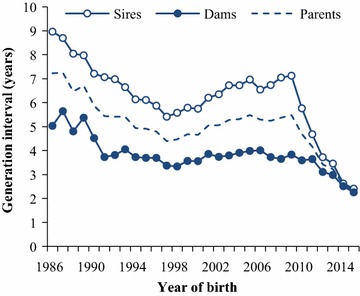
Generation interval for bull sires, bull dams and bull parents by year of birth

Pedigrees were extracted from the database of CRV and extended with publicly available data [25]. The total pedigree comprised 46,232 animals. Complete generation equivalents (CGE) were computed as the sum of (1/2)^*n*^ over all known ancestors, with *n* being the generation number of a given ancestor. The average CGE increased from 9.6 in 1986 to 17.0 in 2015 and was equal to 13.3 when calculated across all years. The average number of completely known generations increased from 4.1 in 1986 to 8.1 in 2015. The generation interval (*L*), i.e. the average age of parents at the birth of the bulls, was computed per year of birth for bull sires and bull dams separately, and for all parents combined (Fig. 2). The *L* decreased during the first decade and then increased slightly until it dropped steeply from 2009 onwards. The initial drop in *L* can be explained by an increased use of young unproven bull sires, which, at the time, was expected to improve genetic gain. However, due to variable gains, the trend changed and, from 1998 onwards, almost exclusively proven bull sires were used. The drop in *L* from 2009 onwards was especially pronounced for bull sires and followed the implementation of GS. The average *L* across the whole 30-year period and for all parents combined was 5.0 years.

Genotype data were provided by CRV and the final dataset comprised 75,538 autosomal SNPs. Bulls were genotyped with the Illumina BovineSNP50 BeadChip (versions v1 and v2) or CRV custom-made 60 k Illumina panel (versions v1 and v2). Genotypes were imputed to ~ 76 k from the different panels, following Druet et al. [26], and haplotypes were constructed with a combination of Beagle [27] and PHASEBOOK [28], by exploiting both familial and population information. Prior to imputation, SNPs with a call rate lower than 0.85, a MAF lower than 0.025 or a difference larger than 0.15 between observed and expected heterozygosity were discarded. SNP positions were obtained from the Btau4.0 genome assembly and SNPs with unknown positions (N = 893) were discarded. The mean physical distance between two consecutive SNPs was 33.7 kb, with density varying substantially across the genome (see Additional file 1: Fig. S1). Black and white (N = 5021) and red and white (N = 1259) bulls were combined in all analyses, because a preliminary check on the mean SNP-based kinship within and between bulls of both groups indicated no major genetic differentiation across the 30-year period.

### Genome-wide diversity

Genome-wide diversity was quantified with genealogical, marker-by-marker and segment-based approaches. Pearson correlation coefficients between genealogical, marker-by-marker and segment-based measures were calculated to compare the different approaches.

#### Genealogical inbreeding and kinship

Genealogical coefficients of inbreeding ($F_{{PED_{i} }}$) and kinship ($f_{{PED_{ij} }}$) were defined as the pedigree-based probabilities that two alleles at a (imaginary) selection-free locus, sampled respectively within individual *i* or between individuals *i* and *j*, were IBD with reference to a base population [4]. Founders in the pedigree were considered as the base population. Both $F_{{PED_{i} }}$ and $f_{{PED_{ij} }}$ were calculated with *calc_grm* [29], according to the algorithms of Sargolzaei et al. [30] and Colleau [31].

#### Marker-by-marker homozygosity and similarity

Marker-by-marker homozygosity ($HOM_{{SNP_{i} }}$) and similarity ($SIM_{{SNP_{ij} }}$) were defined as the probabilities that two alleles at a random SNP, which were sampled respectively within individual *i* or between individuals *i* and *j*, were IBS. The $HOM_{{SNP_{i} }}$ was obtained as the proportion of SNPs for individual *i* that were homozygous. The $SIM_{{SNP_{ij} }}$ was determined according to Malécot [16]:

$$SIM_{{SNP_{ij} }} = \frac{{\mathop \sum \nolimits_{k = 1}^{{n_{SNP} }} \left( {I_{11,k} + I_{12,k} + I_{21,k} + I_{22,k} } \right)}}{{4n_{SNP} }},$$

where $n_{SNP}$ is the total number of markers, $I_{xy,k}$ is an indicator variable that was set to 1 when allele *x* of individual *i* and allele *y* of individual *j* at marker *k* were IBS, and to 0 otherwise. Note that the $SIM_{{SNP_{ij} }}$ is equivalent to VanRaden’s genomic relationship *G*_*ij*_ [32] when allele frequencies of 0.5 are used in the computation of *G*_*ij*_ (except for the scale; see Additional file 1 of Eynard et al. [33] for derivation). Since self-similarities $\left( {SIM_{{SNP_{ii} }} = \frac{1}{2}\left[ {1 + HOM_{{SNP_{i} }} } \right]} \right)$ were included, the average similarity in a given cohort was also equivalent to the expected homozygosity in that cohort (i.e. the average sum of squared allele frequencies, $p^{2} + q^{2}$, across all SNPs).

#### Segment-based inbreeding and kinship

Segment-based inbreeding ($F_{{ROH_{i} }}$) was defined as the proportion of the genome of individual *i* that was covered by long uninterrupted homozygous segments. Such regions of homozygosity (ROH) were detected by moving SNP by SNP across chromosomes and testing potential ROH against predefined criteria. The following criteria were used to define a ROH: (1) a minimum physical length of 3.75 Mb, (2) a minimum of 38 consecutive homozygous SNPs (no heterozygous calls allowed), and (3) a maximum gap of 500 kb between two consecutive SNPs. The minimum length of 3.75 Mb was chosen to match the pedigree depth. Given the genetic distance of approximately 1 cM per Mb [34] and the average length of 1/2*G* M for ROH derived from a common ancestor *G* generations ago [19], the $F_{{ROH_{i} }}$ was expected to capture inbreeding over 13.3 ancestral generations (corresponding to the CGE of the pedigree). The latter two criteria were used to prevent calling of (potentially false positive) ROH in regions with low SNP density. The $F_{{ROH_{i} }}$ was calculated as the fraction of the autosome in ROH [17]:

$$F_{{ROH_{i} }} = \frac{{\mathop \sum \nolimits_{m = 1}^{{n_{{ROH_{i} }} }} l_{{ROH_{i,m} }} }}{{l_{a} }},$$

where $n_{{ROH_{i} }}$ is the total number of ROH in individual $i$, $l_{{ROH_{i,m} }}$ is the length of the $m$th ROH and $l_{a}$ is the length of the autosome covered by SNPs (i.e. the autosome length minus the summed length of gaps longer than 500 kb).

Segment-based kinship ($f_{{SEG_{ij} }}$) was defined as the expected $F_{ROH}$ for an offspring of individuals $i$ and $j$. Shared segments were identified by moving SNP by SNP across every possible pair of chromosomes, with one homolog of individual $i$ and one of $j$, and testing potential segments against predefined criteria. The same criteria were used as for calling ROH. The $f_{{SEG_{ij} }}$ was computed following de Cara et al. [18]:

$$f_{{SEG_{ij} }} = \frac{{\mathop \sum \nolimits_{m = 1}^{{n_{{SEG_{ij} }} }} \mathop \sum \nolimits_{{x_{i} }}^{2} \mathop \sum \nolimits_{{y_{j} }}^{2} l_{{SEG_{ij,m} }} }}{{4l_{a} }},$$

where $n_{{SEG_{ij} }}$ is the total number of shared segments between individuals $i$ and $j$, $l_{{SEG_{ij,m} }}$ is the length of the $m$th shared segment measured over homolog $x$ of individual $i$ and homolog $y$ of individual $j$ and $l_{a}$ is the length of the autosome covered by SNPs.

#### Rate of change and effective population size

For each genome-wide parameter, the annual rate of change ($\Delta x_{y}$) for the 1986–2015 period was obtained as the opposite of the slope of the regression of $LN(1 - \bar{x})$ on year of birth, where $\bar{x}$ equalled the average of the parameter in a given year [35]. The annual rate was multiplied by $L$ to obtain the rate per generation ($\Delta x_{gen}$) and, subsequently, the effective population size ($N_{e} = 1/(2\Delta x_{gen})$). To investigate trends over time, $\Delta x_{y}$ and $\Delta x_{gen}$ were also calculated for 5-year periods, taking changes in $L$ into account.

### Region-specific inbreeding

Accumulation of inbreeding across the genome over time was evaluated with ROH-based positional inbreeding coefficients. For every marker $k$ in bull $i$, a positional inbreeding coefficient ($F_{{ROH_{i,k} }}$) was set to 1 when $k$ was encompassed by a ROH, and to 0 otherwise, following Kim et al. [36]. The $F_{{ROH_{k} }}$ per 5-year period was then calculated as the fraction of bulls born in that period for which $k$ was encompassed by a ROH.

### Changes in allele frequency and putative selected regions

Changes in allele frequency were computed as $\Delta p = p_{t} - p_{0}$, where $p_{t}$ and $p_{0}$ were the frequency in the last (2011–2015) and first (1986–1990) 5-year periods, respectively. Since the average $L$ was 5.0 years, the $\Delta p$-values were based on approximately five generations of drift and selection. To identify putative selected regions, the observed $\Delta p$-values were compared to those expected under pure genetic drift. The $\Delta p$-distribution under pure drift was obtained by gene dropping [37]. In each simulated gene drop, alleles for a single SNP were randomly assigned to founders and subsequently dropped through the pedigree following Mendelian principles (i.e. random sampling). To ensure a wide spectrum of $p_{0}$-values, founder minor allele frequencies (MAF) ranging from 0.5 to 50% were simulated. Realised $p_{0}$-values were classified into 100 MAF-classes, ranging from 0.0–0.5% to 49.5–50.0%, and the drift distribution per MAF-class was obtained based on 3000 replicates. Observed absolute $\Delta p$-values above the 99.9% threshold (*P *< 0.001) of the empirical gene drop distribution were considered indicative of selection. To visualise systematic changes over the erratic pattern of individual SNPs, the moving average of 31 adjacent absolute $\Delta p$-values was plotted against the physical position of the central SNP.

Genomic regions with an excess of putative selected SNPs were considered as putative selected regions. For the key regions of interest, we investigated which QTL were known in these regions, using AnimalQTLdb [38]. The complete CattleQTLdb, which contains 99,675 QTL, was first filtered; QTL mapped to chromosome X (N = 25,589), reported for non-HF breeds (N = 23,468) and/or with unknown start and end positions (N = 1737) were discarded. In addition, QTL associated to traits that were not clearly related to the Dutch-Flemish breeding bull-selection index, such as specific milk fatty acids or carcass traits, were removed (N = 21,195). This resulted in a final list of 27,662 QTL, associated to 61 traits classified in five trait categories: production (INET), conformation (CONF), longevity (LONG), reproduction (REPR) and udder health (UH). The final list of traits and number of QTL per trait and trait category is included in Table S1 (Additional file 2: Table S1).

Changes in allele frequency were also computed within each 5-year period as $\Delta p = p_{t} - p_{0}$, with $p_{t}$ and $p_{0}$ being the frequencies in the last and first year of the period, respectively (e.g. $\Delta p = p_{1990} - p_{1986}$). Correlation coefficients between the $\Delta p$-values of the different 5-year periods were calculated to investigate the direction of changes in allele frequency over time.

## Results

### Genome-wide diversity

Descriptive statistics for all six genome-wide parameters are shown in Table 1. The average genealogical inbreeding and kinship were 5.2 and 6.5%, respectively. Segment-based coefficients were on average ~ 1.5% higher than genealogical coefficients. As expected, IBS coefficients showed a higher mean (64.4% for $HOM_{SNP}$ and 64.8% for $SIM_{SNP}$), lower SD and lower CV than IBD coefficients. For all kinship parameters, the mean was considerably higher than the median, which was indicative of the right-skewedness of the underlying distributions that was due to the inclusion of self-kinships.

**Table 1:** Descriptive statistics for genome-wide inbreeding and kinship parameters in all years combined

Parameter	N	Mean	SD	Median	Min.	Max.	CV
$F_{PED}$	6280	5.206	2.250	5.097	0.000	17.876	0.432
$F_{ROH}$	6280	6.750	2.892	6.427	0.669	25.378	0.429
$HOM_{SNP}$	6280	64.360	1.177	64.223	58.425	71.838	0.018
$f_{PED}$	1,470,166	6.538	4.581	5.685	0.267	58.938	0.701
$f_{SEG}$	1,470,166	7.987	4.609	7.143	0.006	62.689	0.577
$SIM_{SNP}$	1,470,166	64.823	1.777	64.473	61.530	85.919	0.027

Values are shown as percentages

*N* number of coefficients, *SD* standard deviation, *Min.* minimum, *Max.* maximum, *CV* coefficient of variation, $F_{PED}$ and $f_{PED}$ genealogical inbreeding and kinship, $F_{ROH}$ and $f_{SEG}$ segment-based inbreeding and kinship, $HOM_{SNP}$ and $SIM_{SNP}$ marker-by-marker homozygosity and similarity

**Fig. 3:**
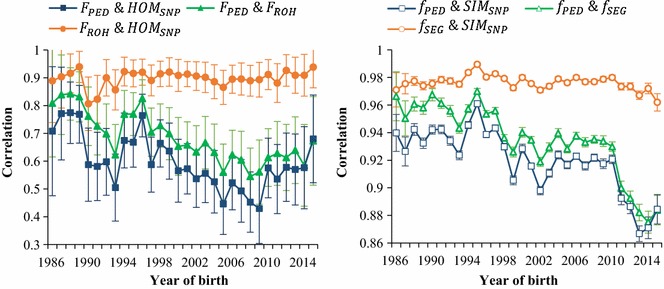
Correlations between different genome-wide estimates of inbreeding (left) and kinship (right) by year of birth. Note the different scales for the y-axes for inbreeding and kinship. Self-kinships were excluded from the computation to remove the influence of the number of bulls per year on the correlations. Error bars represent ± 2 standard errors. $F_{PED}$ and $f_{PED}$: genealogical inbreeding and kinship; $F_{ROH}$ and $f_{SEG}$: segment-based inbreeding and kinship;$HOM_{SNP}$ and $SIM_{SNP}$: marker-by-marker homozygosity and similarity

Pearson correlation coefficients between different genome-wide estimates of inbreeding and kinship per year of birth are shown in Fig. 3. Correlations between kinship parameters were considerably higher than those between inbreeding coefficients. Over all years, the highest correlations were found between the genomic parameters (on average 0.90 for $HOM_{SNP}$ with $F_{ROH}$ and 0.98 for $SIM_{SNP}$ with $f_{SEG}$) and the lowest between the marker-by-marker and genealogical estimates (on average 0.60 for $HOM_{SNP}$ with $F_{PED}$ and 0.92 for $SIM_{SNP}$ with $f_{PED}$). Correlations between genomic parameters remained relatively constant over years, whereas correlations between pedigree and genomic parameters decreased over time. For example, the correlation between $f_{SEG}$ and $f_{PED}$ decreased from 0.97 in 1986 to 0.88 in 2015. This divergence could be explained by the accumulation of Mendelian sampling variation over time, which is captured by genomic information, but not by pedigree data. When more generations are included in the calculation of $f_{PED}$, more sampling events are unaccounted for and $f_{PED}$ is likely to deviate more from the realised genomic relationship. Correlations between pedigree and genomic inbreeding parameters seemed to increase slightly from 2009 onwards. However, this increase could also be due to random fluctuations, as the standard errors for inbreeding correlations were rather large (Fig. 3).

**Fig. 4:**
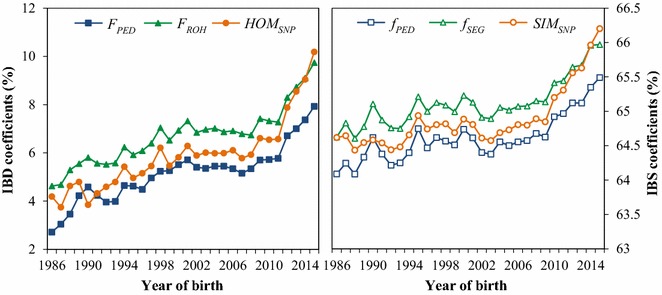
Average genome-wide inbreeding (left) and kinship (right) by year of birth. Coefficients of IBD ($F_{PED}$, $F_{ROH}$, $f_{PED}$, $f_{SEG}$) and IBS ($HOM_{SNP}$, $SIM_{SNP}$) are shown on the primary and secondary y-axis, respectively. $F_{PED}$ and $f_{PED}$: genealogical inbreeding and kinship; $F_{ROH}$ and $f_{SEG}$: segment-based inbreeding and kinship;$HOM_{SNP}$ and $SIM_{SNP}$: marker-by-marker homozygosity and similarity

Roughly, genome-wide inbreeding increased from 1986 to 2000, remained rather constant for a decade and then steeply increased from 2011 onwards (Fig. 4). Genome-wide kinship levels fluctuated more, but also increased from 1986 to 2000, temporarily dropped and then remained rather constant until a steep increase from 2009 onwards.

Genome-wide rates of change per year and per generation for the 1986–2015 period are shown in Table 2. Estimates of $N_{e}$ computed from $\Delta F_{PED}$, $\Delta F_{ROH}$ and $\Delta HOM_{SNP}$ were equal to 79, 75 and 69, respectively. Rates of kinship were lower than rates of inbreeding, with a $N_{e}$ estimated from $\Delta f_{PED}$, $\Delta f_{SEG}$ and $\Delta SIM_{SNP}$ of 102, 100 and 91, respectively. The difference between inbreeding and kinship rates was largely due to the relatively high kinship levels in early years (Fig. 4). In fact, the average kinship at the beginning of the period was more than two generations ahead of the average inbreeding, while a difference of a single generation is expected for a randomly mating population.

**Table 2:** Genome-wide rates of change and effective population size (${N}_{{e}}$) for the period 1986–2015

Parameter	Rate of change (%) per		
	Year	Generation	${N}_{{e}}$
$F_{PED}$	0.1280	0.6354	78.67
$F_{ROH}$	0.1342	0.6663	75.04
$HOM_{SNP}$	0.1462	0.7261	68.86
$f_{PED}$	0.0984	0.4887	102.31
$f_{SEG}$	0.1001	0.4991	100.19
$SIM_{SNP}$	0.1108	0.5502	90.88

$F_{PED}$ and $f_{PED}$ genealogical inbreeding and kinship, $F_{ROH}$ and $f_{SEG}$ segment-based inbreeding and kinship, $HOM_{SNP}$ and $SIM_{SNP}$ marker-by-marker homozygosity and similarity

**Fig. 5:**
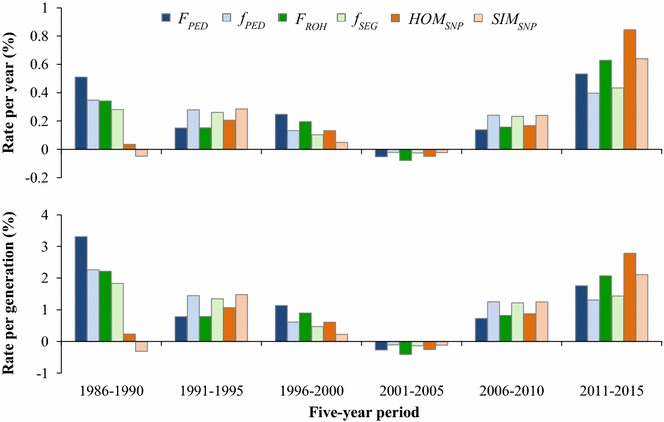
Rate of change per year (top) and generation (bottom) for genome-wide parameters within 5-year periods. $F_{PED}$ and $f_{PED}$: genealogical inbreeding and kinship; $F_{ROH}$ and $f_{SEG}$: segment-based inbreeding and kinship;$HOM_{SNP}$ and $SIM_{SNP}$: marker-by-marker homozygosity and similarity

Rates of inbreeding and kinship were also computed for periods of 5 years, accounting for the change in $L$ over time. Both rates per year and per generation decreased over the first four periods, were slightly negative between 2001 and 2005 and increased in the last two periods (Fig. 5). In the 2011–2015 period, rates of $\Delta F_{PED}$, $\Delta F_{ROH}$ and $\Delta HOM_{SNP}$ were as high as 1.8, 2.1 and 2.8% per generation, respectively. Rates of change were very similar across the three approaches, except in the first, third and last periods. In the 1986–1990 period, the $\Delta HOM_{SNP}$ and $\Delta SIM_{SNP}$ were close to zero as a result of large fluctuations in IBS levels (Fig. 4). In this period, $\Delta F_{PED}$ was also relatively high (i.e. 1% higher per generation than $\Delta F_{ROH}$). In the 1996–2000 period, genealogical rates of inbreeding were slightly higher (0.1–0.2% higher per generation) than segment-based rates, which, in turn, were slightly higher (0.2–0.3%) than marker-based rates. In the last period, which showed almost no fluctuations, marker-based rates were considerably higher (0.7% per generation) than segment-based rates, which were in turn slightly higher (0.3% for $\Delta F$ and 0.1% for $\Delta f$) than genealogical rates of inbreeding.

### Region-specific inbreeding

**Fig. 6:**
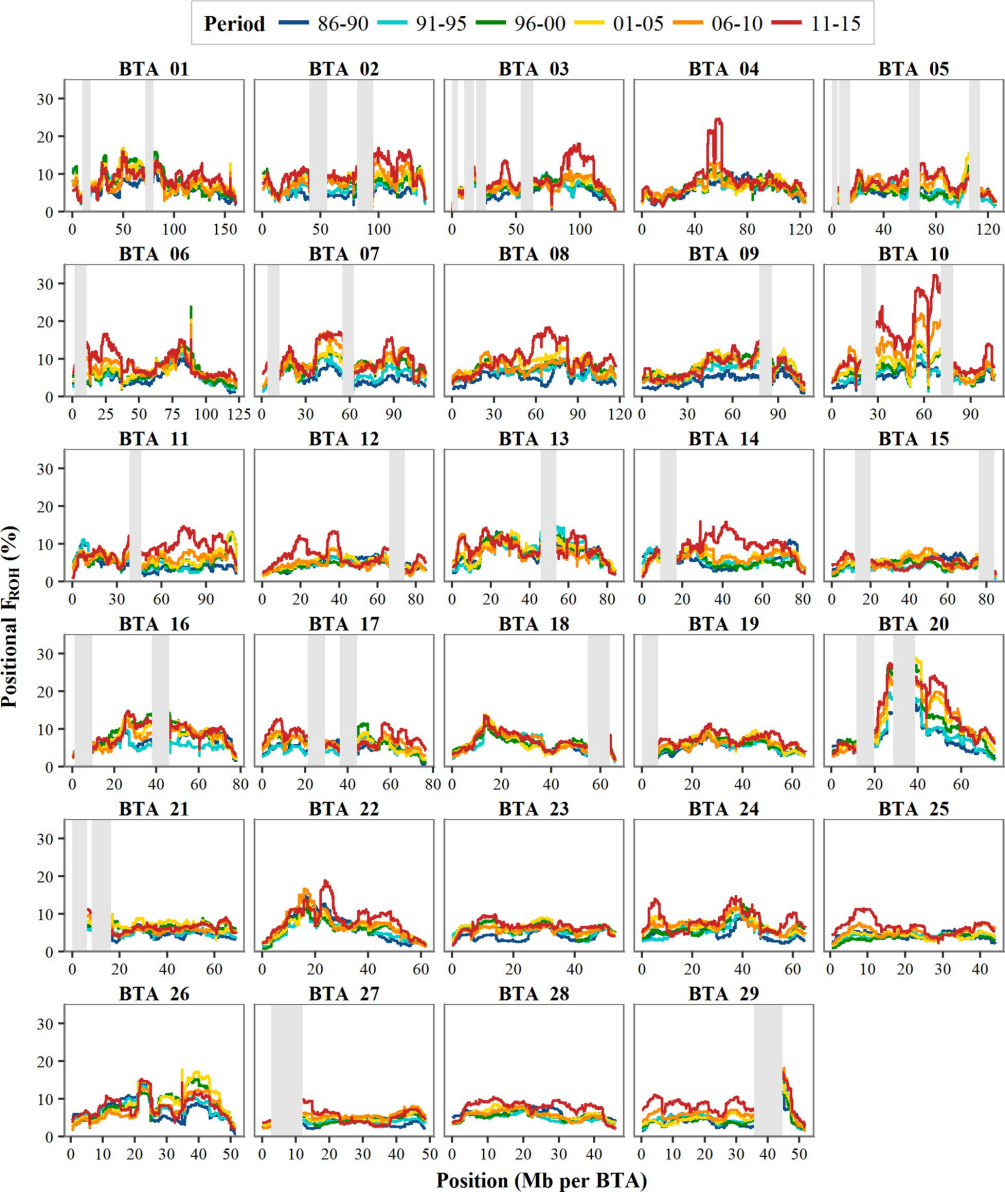
Positional inbreeding coefficients (${F}_{{{ROH}}}$) per 5-year period between 1986 and 2015. Grey bars cover gaps between consecutive markers of > 500 kb (with an additional 3.75 Mb on both sides of the gap). BTA: *Bos taurus* autosome. Note that the scale of the x-axis differs between chromosomes

Accumulation of inbreeding across the genome was evaluated with ROH-based positional inbreeding coefficients ($F_{{ROH_{k} }}$). Substantial heterogeneity was observed in the levels of $F_{{ROH_{k} }}$ over time (Fig. 6). There were, among others, regions with a continuous increase in inbreeding (e.g. the peaks on BTA10), regions with an increase followed by a decrease (e.g. around 40 Mb on BTA26) and regions with a constant inbreeding level over time (e.g. BTA18). Particularly striking was the strong increase in $F_{{ROH_{k} }}$ in the last period for various regions (e.g. around 55 Mb on BTA4, around 40 Mb on BTA14 and around 25 Mb on BTA22). Overall, BTA10 showed the most prominent increase in $F_{{ROH_{k} }}$, from 5% in the 1986–1990 period to 20–30% in the 2011–2015 period at the peak regions. BTA20 also showed regions with a $F_{{ROH_{k} }}$ of 20–30% in the 2011–2015 period, but these peaks had already a higher $F_{{ROH_{k} }}$ at the start of the 30-year period (of 10–15%). Within the high peak on BTA10, there was a remarkable trough near 62.5 Mb, which could be due to incorrect SNP positions on the reference genome Btau4.0 (the 12 SNPs in this region were mapped near 71.5 Mb on UMD3.1). The trough within the peak on BTA4, near 55 Mb, might also be the result of incorrect SNP positions, although for this region there was no inconsistency between Btau4.0 and UMD3.1 positions.

### Changes in allele frequency and putative selected regions

**Fig. 7:**
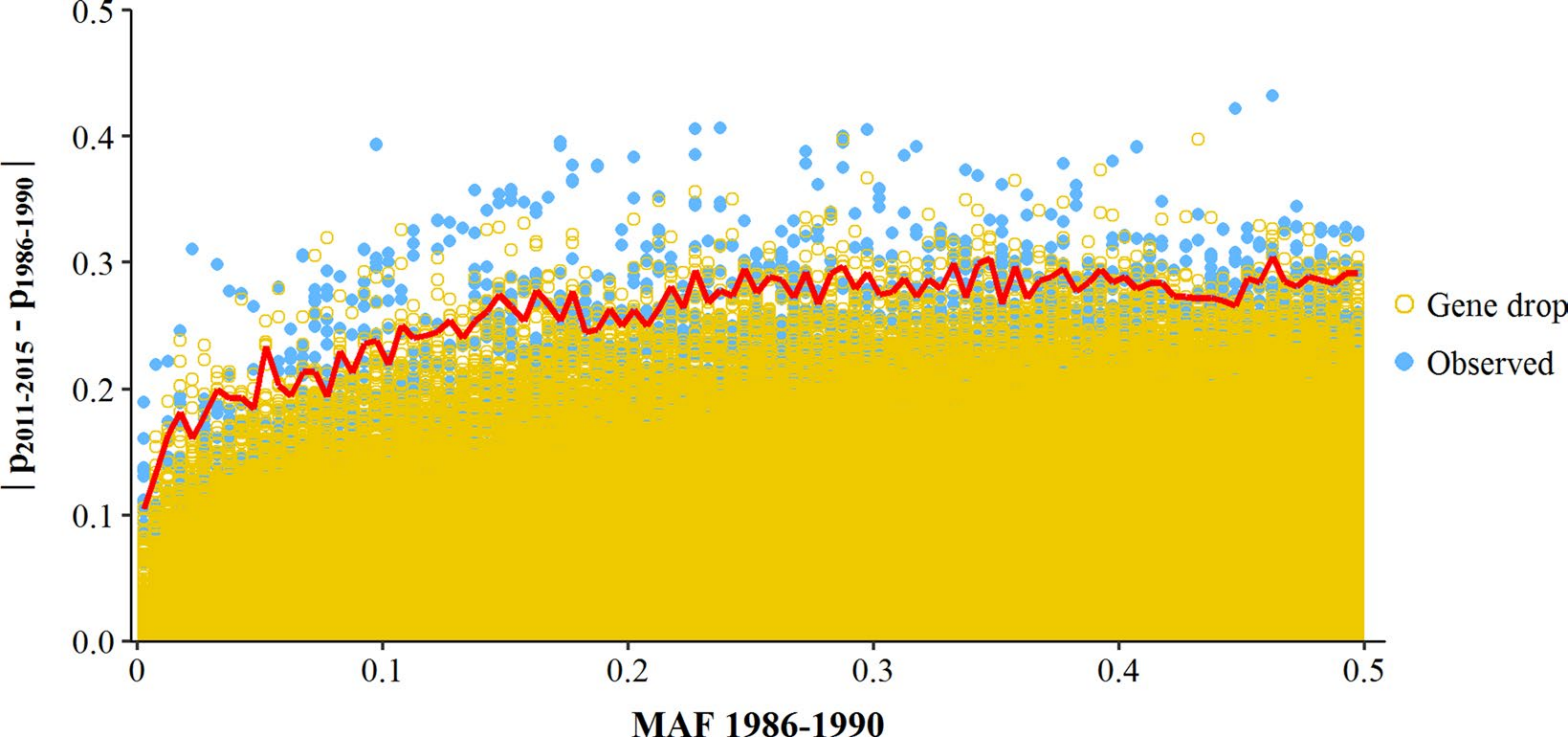
Absolute allele frequency changes from 1986–1990 to 2011–2015 ($\left| {{p}_{2011 - 2015} - {p}_{1986 - 1990} } \right|$) observed in data and gene drop. Changes are shown for different minor allele frequencies (MAF) in the 1986–1990 period, using MAF-classes of 0.5% (e.g. 0.0–0.5%). The red line represents the 99.9%-threshold of the gene drop distribution per MAF class

**Fig. 8:**
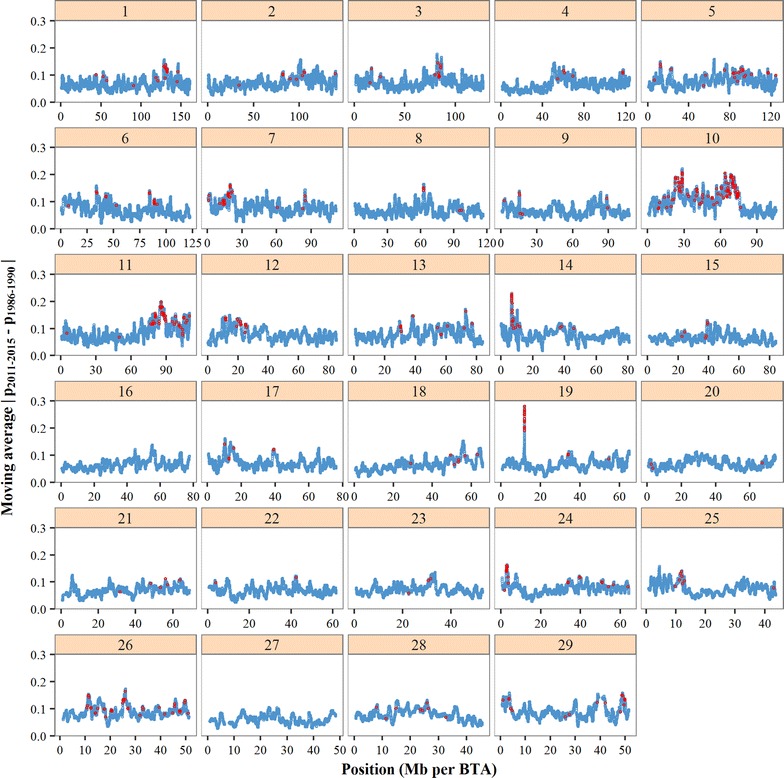
Moving average of absolute changes in allele frequency from the 1986–1990 to the 2011–2015 period ($\left| {{p}_{2011 - 2015} - {p}_{1986 - 1990} } \right|$). Moving average is based on 31 SNPs. The SNPs in red (N = 490) have an allele frequency change above the 99.9%-threshold of the gene drop distribution (see Fig. 7). *BTA* Bos taurus autosome

Absolute changes in allele frequencies from the 1986–1990 period to the 2011–2015 period, $\left| {\Delta p} \right|$, were compared with those expected from gene dropping (Fig. 7). Many SNPs showed higher $\left| {\Delta p} \right|$-values than would be expected under pure genetic drift. For example, there were 6835 SNPs (9.05% of the total number) and 490 SNPs (0.65% of the total number) with a $\left| {\Delta p} \right|$ above the 95%- and 99.9%-thresholds of the gene drop distribution, respectively. The SNPs above the 99.9%-threshold were considered indicative of selection and, although they were spread across the whole genome, these SNPs were generally located in peaks of high $\left| {\Delta p} \right|$ (Fig. 8). In line with the pattern observed for $F_{{ROH_{k} }}$ (Fig. 6), BTA10 showed the highest $\left| {\Delta p} \right|$ on average, with two wide peaks enriched with putative selected SNPs. However, on BTA20 no clear peak was observed and only three putative selected SNPs were detected. In contrast, BTA19 showed a narrow peak for $\left| {\Delta p} \right|$ that was not present in Fig. 6. This could be explained by the extremely high SNP density in this region (see Additional file 1: Fig. S1), which caused the moving average of 31 $\left| {\Delta p} \right|$-values to be based on a region of only 50 kb (while for ROH only regions longer than 3.75 Mb were considered).

For 11 regions that were enriched with putative selected SNPs, we investigated whether QTL were known in these regions (Table 3). In general, the putative selected regions were large and overlapped with many QTL of different trait categories. Across all regions combined, there was a relatively large number of QTL for conformation traits and relatively few for production traits, when compared to QTL reported for the complete autosome. The relatively low fraction of QTL for production-traits could be explained by the fact that 39% of all production-QTL in the AnimalQTLdb are located on BTA14, whereas only a single short region on this chromosome was identified in this study as a putative selected region.

**Table 3:** Putative selected regions based on changes in allele frequency from the 1986–1990 period to the 2011–2015 period and fraction of known QTL mapped to these regions per trait category

BTA	Start–end position (Mb)	${n}_{{{QTL}}}$	Fraction of QTL per trait category (%)				
			INET	CONF	LONG	REPR	UH
1	128.0–133.0	83	4	34	8	46	8
3	80.0–86.0	68	6	47	7	31	9
7	10.5–22.0	169	7	36	19	31	8
10	19.0–29.0	43	37	30	5	23	5
10	60.0–75.0	111	9	76	2	9	5
11	76.0–89.5	342	15	70	1	14	1
12	19.0–26.0	42	29	21	7	38	5
14	6.0–8.0	44	91	2	2	2	2
19	11.5–12.0	1	0	0	0	0	100
24	1.5–4.0	26	35	27	8	27	4
26	25.0–27.5	30	17	50	3	23	7
Total putative selected regions		959	17	51	6	22	4
Complete autosome		27,662	38	25	8	26	3

QTL were included when reported in AnimalQTLdb [38]. QTL were classified into five trait categories: INET (production index), CONF (conformation), LONG (longevity), REPR (reproduction) or UH (udder health). See Additional file 2 for classification of traits

To evaluate the direction of allele frequencies over time, correlation coefficients between the $\Delta p$ within different 5-year periods were calculated (Table 4). Except for the correlation between the 1996–2000 and 2011–2015 periods, all correlations were significantly different from 0 (*P* < 0.0001). Correlation coefficients for any two consecutive periods were positive (ranging from 0.08 to 0.26), except for the transition from the 1996–2000 period to the 2001–2005 period (− 0.09).

**Table 4:** Correlations between allele frequency changes (e.g. ${p}_{1990} - {p}_{1986}$) within different 5-year periods between 1986 and 2015

Period	86–90	91–95	96–00	01–05	06–10
91–95	0.094				
96–00	0.089	0.082			
01–05	− 0.062	− 0.130	− 0.094		
06–10	− 0.028	− 0.113	− 0.087	0.092	
11–15	0.040	− 0.041	0.001	− 0.070	0.264

Standard errors of correlations ranged from 0.0004 (for 1996–2000 with 2011–2015) to 0.0035 (for 2006–2010 with 2011–2015)

## Discussion

In this study, we evaluated genetic diversity across the genome of HF AI bulls from 1986 to 2015. An important objective was to investigate whether major changes in the Dutch-Flemish HF breeding program were accompanied by changes in diversity trends. We used genealogical, marker-by-marker and segment-based approaches to compare trends in expected IBD, IBS and realised IBD.

Genome-wide rates of inbreeding and kinship and corresponding estimates of $N_{e}$ computed over the 1986–2015 period were similar to those previously reported for HF populations. Genealogical and genomic estimates of $N_{e}$ for HF populations in Australia, Canada, Denmark, Spain, Ireland and the United States of America for (parts of) the 1975–2013 period range from 49 to 127 [5–7, 39, 40]. A similar $N_{e}$ across countries is expected, due to the extensive exchange of genetic material. In spite of the global connectedness of the breed, there is some degree of genetic differentiation across countries [7, 41].

Genome-wide diversity trends showed two breakpoints. The first occurred around 2000, after which levels and rates of inbreeding and kinship temporarily dropped (Figs. 4 and 5). The second occurred around 2010, after which inbreeding and kinship steeply increased. Both breakpoints coincided with major changes in the Dutch-Flemish breeding program.

The drop in inbreeding and kinship around 2000 followed a shift in breeding goal composition and the introduction of OCS. Although the Dutch-Flemish bull selection index has changed continuously over time, the major shift took place around 2000, when longevity, udder health and reproductive traits were added to the index within a few years’ time (Table 5). The inclusion of a wide range of traits at that time resulted in a more diverse set of bulls with high estimated breeding values (EBV) and thereby contributed to the (temporary) drop in inbreeding and kinship. From 2000 onwards, pedigree-based OCS has been used to select bull-parents in the breeding program and restrict $\Delta F$ and $\Delta f$. However, the effect of OCS will have been limited due to practical difficulties. One such difficulty is that, in practice, not all candidates with allocated contributions are available for breeding. Another difficulty is that OCS considers all candidates at a single moment in time, while selection decisions in the breeding program are made on a daily basis. In spite of these difficulties, the use of OCS will have restricted $\Delta F$ and $\Delta f$ and its introduction will have contributed to the observed drop around 2000. A drop in $\Delta F$ and $\Delta f$ around 2000 was also observed in the Canadian and Danish HF populations [5, 40], although less pronounced than the drop in the current study. In these other HF populations, OCS was not (yet) introduced at that time. Stachowicz et al. [5] suggested that the drop in the Canadian population may be due to an increased awareness and the introduction of average relationship values (R-values) by the Canadian Dairy Network around 2000.

**Table 5:** Relative emphasis of trait categories in the Dutch-Flemish bull selection index over time

Year	Index	Relative emphasis of trait category (%)					References
		INET	CONF	LONG	REPR	UH	
1980	INET	100	–	–	–	–	[42]
1989	Stiersom	67	33	–	–	–	[42, 43]
1999	DPS	67	–	33	–	–	[44]
2003	DPS	58	–	26	12	4	[23]
2007	NVI	40	27	8	16	9	[45]
2012	NVI	26	30	11	19	14	[46]

Note that the relative emphasis of trait categories may not be calculated consistently across references

*INET* production index combining milk, fat and protein yield, *CONF* conformation traits, i.e. conformation of udder, legs, muscling and/or general stature, *LONG* longevity or durability, *REPR* reproductive traits including fertility and birth traits, *UH* udder health or somatic cell count

The steep increase in inbreeding and kinship rates around 2010 coincided with the implementation of GS. From the 2006–2010 period to the 2011–2015 period, there was a two- to four-fold increase in the annual rate of inbreeding. Rates per generation were also considerably higher since the implementation of GS, although the difference was less pronounced due to the decrease in $L$. Rates of $\Delta F_{PED}$, $\Delta F_{ROH}$ and $\Delta HOM_{SNP}$ between 2011 and 2015 were as high as 1.8, 2.1 and 2.8% per generation, respectively (Fig. 5). These rates correspond to an $N_{e}$ of 18, 24 and 28, respectively. Rates of kinship were lower than rates of inbreeding, but were also well above the rates of 0.5–1% per generation recommended for livestock populations [47, 48]. The high rates per generation were rather unexpected, because, in theory, GS reduces $\Delta F_{gen}$ for a given genetic gain compared to traditional best linear unbiased prediction (BLUP) selection, by predicting Mendelian sampling terms and reducing the co-selection of sibs [15, 49].

Estimates of inbreeding and kinship rates in real life HF GS schemes are still scarce. Rodríguez-Ramilo et al. [6] recently evaluated genealogical and genomic inbreeding and kinship trends in the Spanish HF population. They reported $N_{e}$ estimates that increased from 74 to 79 in the 1980–1999 period to 95–101 in the 2000–2013 period as a consequence of a reduction in $L$, but did not evaluate the years with GS separately [6]. For the global HF population, Miglior and Beavers [50] indicated that, although the number of AI bull sires has increased since GS, the number of sires that father 50% of the AI bulls has remained relatively constant. In North-American AI bulls, they also reported an increase of 1% in $F_{PED}$ from 2011 to 2012 [50], which is in line with the 0.94% increase in the current study (Fig. 4).

An important factor that contributes to the accumulation of kinship in GS schemes is the relationship of selection candidates with the reference population. In GS, genomic EBV (GEBV) are computed from the effects of SNPs, which are estimated in a reference population of individuals with known genotypes and phenotypes [24]. The accuracy of an individual’s GEBV is strongly affected by the genetic relationship between the individual and the reference population [51–53]. Pszczola et al. [51] indicated that the average squared relationship of a candidate with the reference population influences especially the accuracy of GEBV. This means, for example, that having a single full sib in the reference population contributes more to a candidate’s GEBV accuracy than having two half-sibs. In general, candidates with a high average squared relationship with the reference population have a more accurate GEBV and are, therefore, more likely to be selected at a young age. This implies that, in a way, genetic variation in the reference population drives variation in selected individuals, which in turn drives variation at the population level. Thus, the composition of the reference population is an essential parameter that requires careful consideration for the management of diversity in the population.

Since the implementation of GS, rates of marker-by-marker homozygosity and similarity have been considerably higher (0.7%) than segment-based rates, which in turn have been slightly higher (0.1–0.3%) than genealogical rates. The higher rate for IBS suggests that relatedness due to distant common ancestors is increasing relatively fast compared to relatedness caused by common ancestors in more recent generations. This could be due to the discordance between the way breeding values are estimated and the way diversity is managed. In the current Dutch-Flemish breeding program, breeding values are predicted with genomic BLUP (GBLUP) and are, thus, based on marker-by-marker similarities weighted by allele frequencies [32]. However, diversity is managed on a genealogical basis by restricting $\Delta f_{PED}$ with OCS. Although the relatively high correlations between $f_{PED}$ and $SIM_{SNP}$ and between $f_{PED}$ and $f_{SEG}$ (Fig. 3) suggest that genomic IBD and IBS can be quite efficiently managed using $f_{PED}$, it is important to revisit this idea in view of OCS. In fact, when OCS is performed with GBLUP and a restriction on $\Delta f_{PED}$, the algorithm will search for selection candidates with a high GEBV and low average $f_{PED}$, thereby putting emphasis on the Mendelian sampling terms that are not captured by the pedigree. As demonstrated by Sonesson et al. [15], the genomic inbreeding rate in such a scenario will substantially exceed the genealogical restriction. In addition, it will result in a IBD profile that is extremely variable across the genome [15]. Thus, controlling diversity at the genomic level should be a priority in the breeding program.

In this study, genomic diversity was characterised with marker-by-marker IBS and segment-based IBD. Both measures have clear advantages and drawbacks with regard to management. The main advantage of using marker-by-marker IBS in OCS is that it is the most effective in conserving diversity [54, 55]. However, a drawback is that it stimulates both alleles of biallelic loci to move to a frequency of 0.5, irrespective of their effects. Thereby, deleterious mutations continue to segregate in the population. To expose and eliminate recessive deleterious mutations, it was suggested to combine OCS with inbred matings [56]. Alternatively, a segment-based IBD matrix can be used in OCS to restrict the increase in recent inbreeding. The rationale behind this approach is that recent inbreeding is more harmful than distant inbreeding, because the latter may have already been purged [57, 58]. In other words, the $F_{ROH}$ is more closely associated with inbreeding depression than $HOM_{SNP}$ [18, 20, 21]. Segment-based metrics can also be used to identify genomic regions that are prone to inbreeding depression [9], although the power of detection is limited by the fact that a single segment can contain multiple shorter haplotypes (or single SNPs) with different effects on the phenotype [9, 59]. Another drawback of the use of ROH and IBD-segments is their arbitrary definition. In this study, we defined the minimum length of IBD segments based on the average CGE of the pedigree, so that both genealogical and segment-based coefficients were expected to capture relatedness over 13.3 ancestral generations. However, the observed segment-based coefficients were on average ~ 1.5% higher than genealogical coefficients. Pedigree skewness, which is not completely accounted for by the CGE, will have contributed to this difference. For example, in an extreme scenario with 20 generations completely known on the sire’s side, but with the dam unknown, the CGE of the offspring equals 10 while the $F_{PED}$ equals 0 by definition. A second factor that strongly influenced the difference between genealogical and segment-based coefficients was the chosen maximum gap length between SNPs. For example, when the maximum gap size was set to 250 kb instead of 500 kb, the segment-based coefficients moved to the same scale as genealogical coefficients. Due to the large effect of such small changes, and the wide variety of criteria used in the literature [36, 60, 61], one should be extremely cautious when comparing segment-based coefficients across studies. A last drawback of the segment-based approach is that it is computationally rather intensive. In spite of these limitations, the use of segment-based metrics is considered a promising tool to determine the effect of inbreeding and, when applied in OCS, to maintain diversity and fitness simultaneously [8, 18, 20].

Selection has played an important role in shaping genetic variation across the HF genome over time. Although the identification of selection footprints was not the primary objective of this study, the regions in Table 3, enriched with ‘significant’ $\left| {\Delta p} \right|$ values, can be considered as putative signatures of selection. The most prominent peaks in $\left| {\Delta p} \right|$ were observed on BTA10 (Fig. 8), which is in line with previously reported selection signatures for HF cattle [36, 62]. Using the extended haplotype homozygosity test (EHH) in German HF cattle, Qanbari et al. [63] detected 161 significant ‘core regions’ under selection, of which 17, 45, and 11 regions were located on BTA2, 10 and 20, respectively. We observed no clear peaks on BTA2. For BTA20, a large region with high $F_{{ROH_{k} }}$ (Fig. 6) was observed, but it showed only small changes in allele frequency (Fig. 8). This could be explained by the fact that $F_{{ROH_{k} }}$ for this region was already high in 1986, which suggests that selection for this region occurred already before the Holsteinisation (the large-scale introduction of HF into national dairy industries in the 1970s and early 1980s). The latter could also explain why this region was identified as a selection signature in various countries [36, 62, 64].

The important role of selection was also apparent from the fact that, in consecutive 5-year periods, allele frequencies changed more often in the same direction than in opposite directions (Table 4). An exception was found when comparing allele frequency changes between the 1996–2000 and 2001–2005 periods, which suggests a change in the direction of selection around this time. Indeed, this change coincided with the implementation of OCS and the major shift in breeding goal composition. To further investigate the change in direction around 2000, a ‘moving correlation’ between $\Delta p$ in the 1996–2000 period and $\Delta p$ in the 2001–2005 period was computed for groups of 51 markers (see Additional file 3: Fig. S2). There were several regions that showed a relatively strong negative correlation (see Additional file 4: Table S2) and which were rather large and harboured many known QTL associated with a wide range of traits. Although some of the identified regions showed a relatively large fraction of QTL related to traits such as reproduction (e.g. the region on BTA1), longevity (e.g. the region on BTA12) or udder health (e.g. the region on BTA13), these findings could not be specifically tied to the changes in breeding goal composition.

Substantial differences in $\left| {\Delta p} \right|$ (Fig. 8) and in the accumulation of $F_{{ROH_{k} }}$ (Fig. 6) were observed across the genome. The emergence of such heterogeneity as a result of selection has been previously investigated in simulation and experimental studies [1, 3, 15]. These studies showed that GS acts more locally across the genome, with more pronounced hitchhiking effects compared to BLUP selection [1, 3, 15]. The striking increase in $F_{{ROH_{k} }}$ from the 2006–2010 period to the 2011–2015 period for various genomic regions (Fig. 6) could be the result of this local selection pressure. The peak regions showing high $F_{{ROH_{k} }}$ remained fairly similar from the 2006–2010 period to the 2011–2015 period, which suggests that GS has not per se changed the regions that are under selection, but has especially increased the intensity of selection at these regions. This hypothesis is supported by the relatively strong positive correlation between $\Delta p$-values in the 2006–2010 period and those in the 2011–2015 period (Table 4).

An important question that should be raised is how heterogeneity in $\left| {\Delta p} \right|$ relates to maximising genetic gain and maintaining genetic diversity. At some loci, it is desirable to increase the frequency of favourable alleles towards fixation. At other loci, a high level of genetic diversity is beneficial, for example to ensure a population’s capacity to combat a wide range of pathogens [65] or to limit inbreeding depression [9]. Thus, it is important to minimise the size of selection footprints [3, 8]. This can be achieved by slowly increasing the frequency of many favourable alleles with small effects, instead of strongly selecting for a few alleles with large effects [15, 66]. Although such an approach will not result in the highest gains in the short term, it will increase the long-term response [67, 68]. To maximise long-term gain further, it is desirable to select for rare favourable alleles, because this will increase the genetic variance [67]. Thus, to optimise long-term response while maintaining diversity, it is recommended to give less weight to SNPs that explain more variance and use a relatively uniform distribution of weights for the computation of GEBV [67, 69].

In general, genomic information offers many opportunities to manage genetic diversity and inbreeding more efficiently in the future (see [8] for a review). Among others, it can be used to control diversity at specific regions [70], select against multiple recessive disorders at the same time [71], estimate dominance effects for a better understanding of inbreeding depression [72], exploit variation in recombination rate across the genome [34] and characterise gene bank collections on the genomic level to optimise these collections and exploit stored material [7]. However, the practical benefit of such new insights and genomic tools in real-life selection schemes has yet to be explored.

## Conclusions

There is substantial heterogeneity in diversity across the genome of HF AI -bulls over time as a result of selection and genetic drift. Trends in genome-wide and region-specific diversity reflect major changes in the Dutch-Flemish breeding program. The introduction of OCS and the shift in breeding goal, which both occurred around 2000, were followed by a temporary drop in inbreeding and kinship and were accompanied by a shift in the direction of changes in allele frequency. The recent introduction of GS around 2010 was accompanied by a substantial increase in the rates of inbreeding and kinship, both per year and per generation and especially at the IBS level. Allele frequencies continued to change in the same direction as before GS. These results provide insight in the effect of breeding practices on diversity across the genome and emphasize the need for efficient management of genetic diversity in HF GS schemes.

## Additional files

**Additional file 1: Fig. S1.** Number of SNPs per bin of 50 kb per *Bos taurus autosome* (BTA).

**Additional file 2: Table S1.** Number of QTL extracted from AnimalQTLdb per trait and trait category.

**Additional file 3: Fig. S2.** Moving correlation (of 51 markers) between changes in allele frequency in the 1996–2000 and 2001–2005 periods.

**Additional file 4: Table S2.** Genomic regions of ≥ 7 Mb with strong negative correlation (*r* ≤ − 0.6) between changes in allele frequency in the 1996–2000 and 2001–2005 periods, and fraction of QTL in these regions per trait category.
