# Supplementary figures and images for: Trends in genome-wide and region-specific genetic diversity in the Dutch-Flemish Holstein–Friesian breeding program from 1986 to 2015
Source: Genet Sel Evol. 2018 Apr 11;50:15. doi: 10.1186/s12711-018-0385-y (PMC5896142; doi:10.1186/s12711-018-0385-y)

#
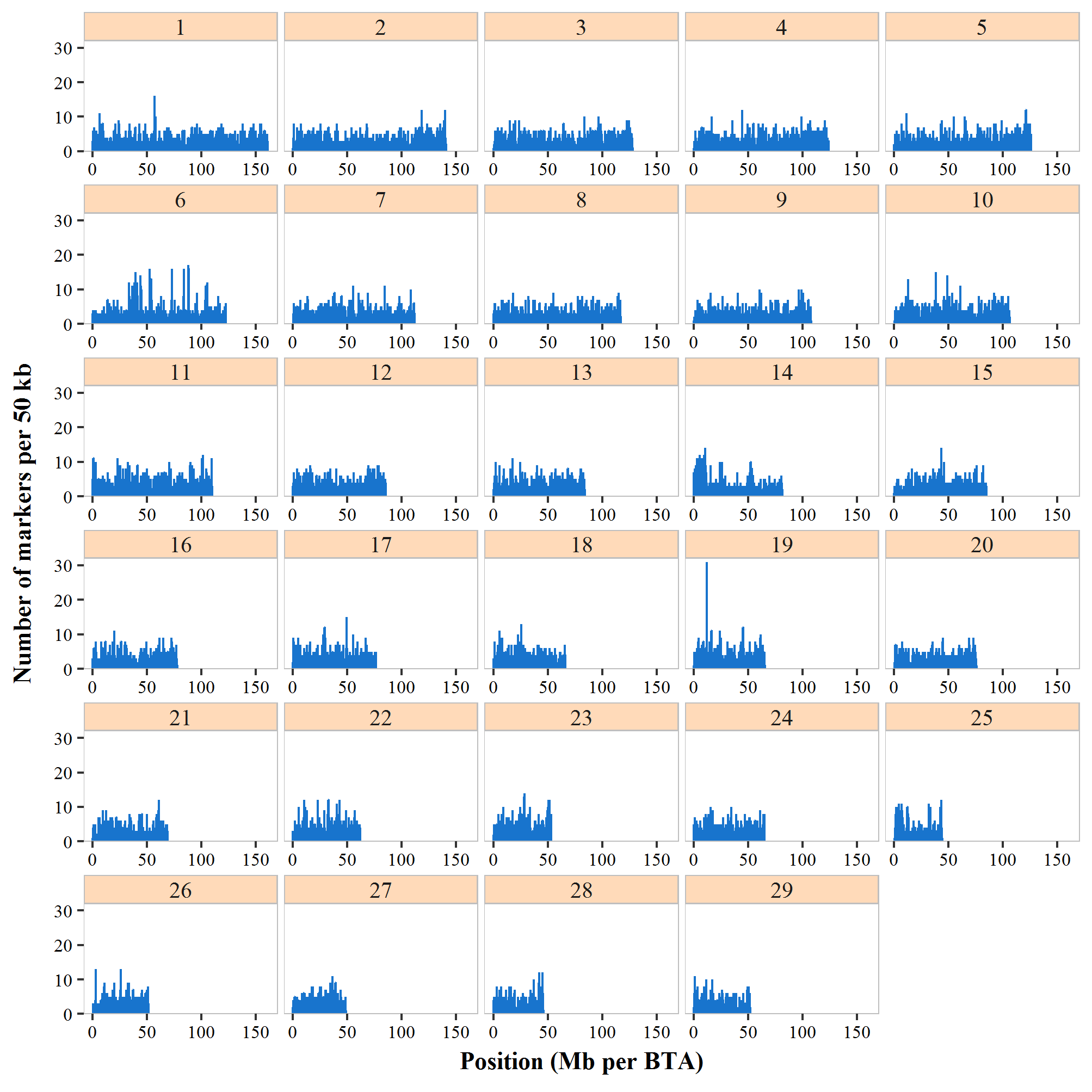

Supplement: Supplementary file 1 — Additional file 1: Fig. S1. Number of SNPs per bin of 50 kb per Bos taurus autosome (BTA). [file 12711_2018_385_MOESM1_ESM.docx]

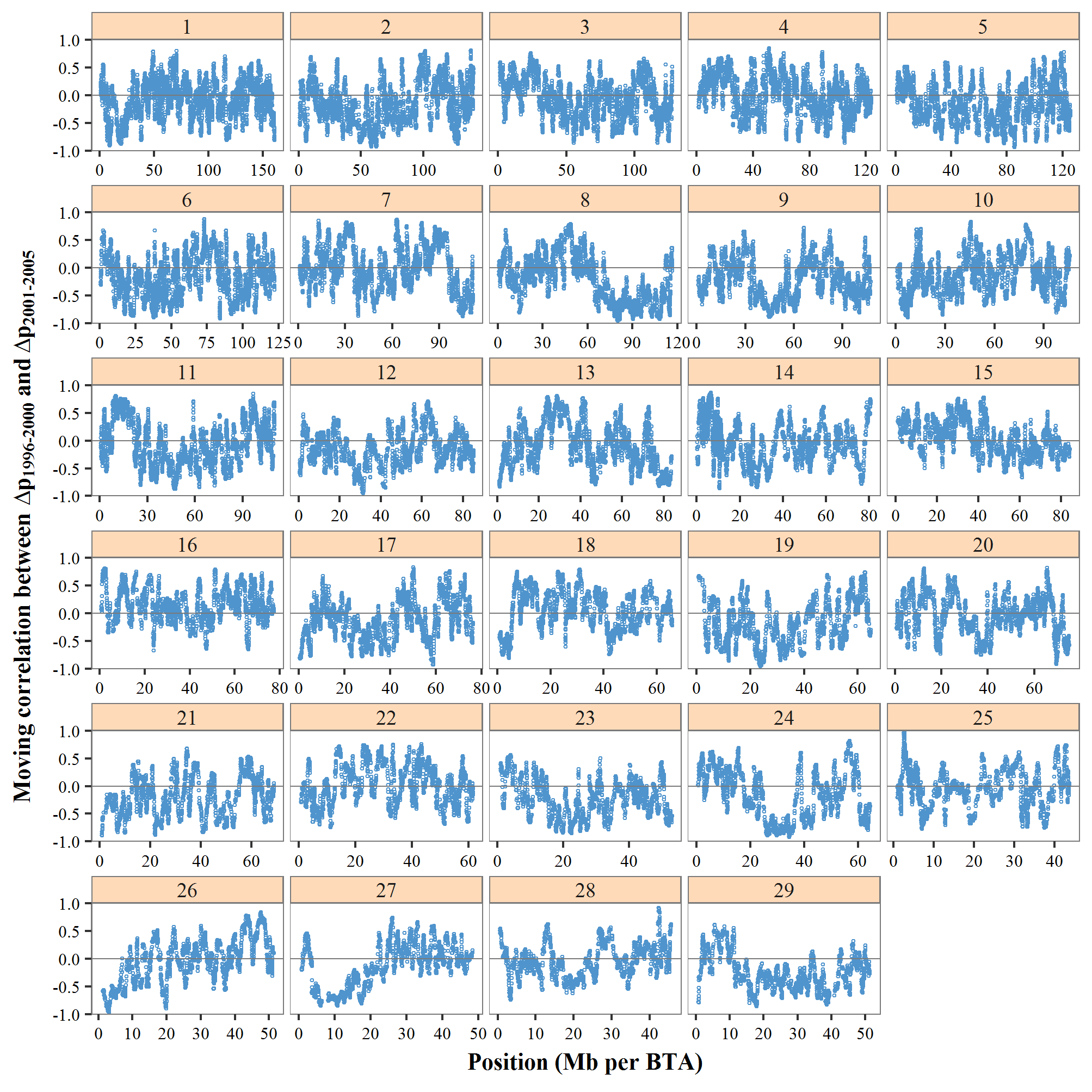

Supplement: Supplementary file 3 — Additional file 3: Fig. S2. Moving correlation (of 51 markers) between changes in allele frequency in the 1996–2000 and 2001–2005 periods. [file 12711_2018_385_MOESM3_ESM.docx]
