# Supplementary material for: Trends in genome-wide and region-specific genetic diversity in the Dutch-Flemish Holstein–Friesian breeding program from 1986 to 2015
Source: Genet Sel Evol. 2018 Apr 11;50:15. doi: 10.1186/s12711-018-0385-y (PMC5896142; doi:10.1186/s12711-018-0385-y)
Supplement: Supplementary file 2 — Additional file 2: Table S1. Number of QTL extracted from AnimalQTLdb per trait and trait category. [file 12711_2018_385_MOESM2_ESM.docx]

| **Category** | **Trait** | **Number of QTL** |
| --- | --- | --- |
| INET | 305-day milk yield | 18 |
|  | Average daily milk yield | 5 |
|  | Milk fat percentage | 2,662 |
|  | Milk fat percentage (daughter deviation) | 228 |
|  | Milk fat percentage (EBV) | 128 |
|  | Milk fat yield | 1,685 |
|  | Milk fat yield (daughter deviation) | 384 |
|  | Milk lactose content | 3 |
|  | Milk lactose yield | 4 |
|  | Milk protein percentage | 2,566 |
|  | Milk protein percentage (daughter deviation) | 236 |
|  | Milk protein yield | 925 |
|  | Milk protein yield (daughter deviation) | 388 |
|  | Milk yield | 876 |
|  | Milk yield (daughter deviation) | 371 |
|  | Milk yield (EBV) | 86 |
|  | Milk yield (ECM) | 21 |
|  | *Total INET* | *10,586* |
| CONF | Body condition score | 18 |
|  | Body depth | 483 |
|  | Conformation score | 12 |
|  | Dairy form | 488 |
|  | Feet and leg conformation | 592 |
|  | Foot angle | 649 |
|  | Hind leg conformation | 4 |
|  | Hoof and leg disorders | 35 |
|  | Rear leg set | 881 |
|  | Stature | 579 |
|  | Teat length | 281 |
|  | Teat number | 5 |
|  | Teat placement | 633 |
|  | Udder attachment | 639 |
|  | Udder cleft | 437 |
|  | Udder depth | 655 |
|  | Udder height | 476 |
|  | Udder structure | 5 |
|  | Udder texture | 10 |
|  | Udder width | 1 |
|  | *Total CONF* | *6,883* |
| LONG | Length of productive life | 2,092 |
|  | Lifetime profit index | 53 |
|  | *Total LONG* | *2,145* |
| REPR | Birth index | 17 |
|  | Calving ease | 894 |
|  | Calving ease (maternal) | 714 |
|  | Calving index | 24 |
|  | Calving interval | 49 |
|  | Calving to conception interval | 46 |
|  | Conception rate | 399 |
|  | Daughter pregnancy rate | 781 |
|  | Fertility index | 45 |
|  | Fertilization rate | 20 |
|  | First service conception | 10 |
|  | Inseminations per conception | 1,611 |
|  | Interval from first to last insemination | 1,463 |
|  | Interval from first to last insemination (EBV) | 1 |
|  | Interval to first estrus after calving | 72 |
|  | Non-return rate | 7 |
|  | Non-return rate (EBV) | 13 |
|  | Stillbirth | 682 |
|  | Stillbirth (maternal) | 377 |
|  | *Total* | *1,744* |
| UH | Clinical mastitis | 77 |
|  | Somatic cell count | 33 |
|  | Somatic cell score | 713 |
|  | *Total* | *823* |
