# Supplementary material for: Trends in genome-wide and region-specific genetic diversity in the Dutch-Flemish Holstein–Friesian breeding program from 1986 to 2015
Source: Genet Sel Evol. 2018 Apr 11;50:15. doi: 10.1186/s12711-018-0385-y (PMC5896142; doi:10.1186/s12711-018-0385-y)
Supplement: Supplementary file 4 — Additional file 4: Table S2. Genomic regions of ≥ 7 Mb with strong negative correlation (r ≤ − 0.6) between changes in allele frequency in the 1996–2000 and 2001–2005 periods, and fraction of QTL in these regions per trait category. [file 12711_2018_385_MOESM4_ESM.docx]

|  | **Start – end**  **position (Mb)** |  |  |  | **Fraction of QTL per trait category (%)** | | | | |
| --- | --- | --- | --- | --- | --- | --- | --- | --- | --- |
| **BTA** |  | $\boldsymbol{r}$ | $\boldsymbol{n}_{\boldsymbol{QTL}}$ |  | **INET** | **CONF** | **LONG** | **REPR** | **UH** |
| 1 | 15.0 – 25.0 | -0.60 | 34 |  | 24 | 26 | 6 | 38 | 6 |
| 2 | 46.0 – 63.0 | -0.60 | 40 |  | 8 | 38 | 10 | 33 | 13 |
| 6 | 16.0 – 24.0 | -0.63 | 51 |  | 20 | 45 | 8 | 25 | 2 |
| 8 | 75.0 – 110.0 | -0.67 | 164 |  | 32 | 24 | 7 | 31 | 5 |
| 9 | 36.0 – 55.0 | -0.60 | 115 |  | 47 | 25 | 5 | 18 | 4 |
| 10 | 3.0 – 10.0 | -0.63 | 39 |  | 10 | 54 | 3 | 33 | 0 |
| 11 | 42.0 – 49.0 | -0.68 | 36 |  | 22 | 53 | 6 | 19 | 0 |
| 12 | 25.0 – 32.0 | -0.65 | 8 |  | 13 | 38 | 13 | 38 | 0 |
| 13 | 77.0 – 84.0 | -0.66 | 19 |  | 26 | 0 | 0 | 21 | 53 |
| 24 | 25.0 – 37.0 | -0.73 | 35 |  | 29 | 31 | 9 | 26 | 6 |
| 26 | 0.0 – 8.0 | -0.68 | 70 |  | 21 | 47 | 10 | 20 | 1 |
| 27 | 4.0 – 13.0 | -0.70 | 70 |  | 33 | 41 | 4 | 21 | 0 |
| *Total* | | *.* | *681* |  | *28* | *34* | *7* | *26* | *5* |
| *Complete autosome* | | *-0.09* | *27,662* |  | *38* | *25* | *8* | *26* | *3* |
| QTL were included when reported in AnimalQTLdb [38]. QTL were classified into five trait categories: INET (production index), CONF (conformation), LONG (longevity), REPR (reproduction) or UH (udder health). See Additional file 2 for classification of traits. | | | | | | | | | |
